# Post-operative Surveillance and Management of Intrahepatic Cholangiocarcinoma Using Circulating Tumor DNA: A Case Report

**DOI:** 10.7759/cureus.55914

**Published:** 2024-03-10

**Authors:** Gary Monroe, Midhun Malla

**Affiliations:** 1 Department of Medicine, West Virginia University School of Medicine, Morgantown, USA; 2 Department of Medicine, University of Alabama at Birmingham School of Medicine, Birmingham, USA

**Keywords:** clinical practice guidelines, cancer surveillance, intrahepatic cholangiocarcinoma, cholangiocarcinoma, circulating tumor dna (ctdna)

## Abstract

Cholangiocarcinomas (CCAs) are a subclass of biliary tract tumors that arise from the epithelial lining of bile ducts. They are subdivided broadly into intra- and extrahepatic CCA, with extrahepatic being the more common. Circulating tumor DNA (ctDNA) is a form of liquid biopsy obtained from dying tumor cells in the peripheral blood. Assays may be tumor-informed or tumor-agnostic, with the former requiring tissue sampling to evaluate detectable mutations present in an individual patient’s tumor. Here we present a case of intrahepatic CCA managed with hepatectomy followed by adjuvant chemotherapy, with subsequent surveillance and management guided by tumor-informed ctDNA.

A 79-year-old female presented to our hospital in December 2019 with three months of postprandial epigastric abdominal pain. Computed tomography (CT) revealed a 5.7 x 5.2 cm left hepatic lobe mass, and surgical pathology confirmed invasive CCA. She underwent left hepatectomy with hepaticojejunostomy one month after presentation and started adjuvant chemotherapy thereafter. She followed us to our cancer center for standard surveillance along with ctDNA. Her tumor markers were within normal limits, and ctDNA was negative until May 2022, when ctDNA was detected, while CA 19-9 remained normal; CT imaging was without evidence of disease. Positron emission tomography-computed tomography (PET-CT) performed in July 2022 revealed local recurrence at the surgical margin, which was confirmed by an endoscopic biopsy. She began gemcitabine-capecitabine chemotherapy in October 2022, completed four cycles followed by chemoradiation therapy, and is currently at her baseline functional status with no detectable radiologic or molecular evidence of disease.

## Introduction

Cholangiocarcinomas (CCAs) are a subclass of biliary tract tumors that arise from the epithelial lining of bile ducts. They are subdivided broadly into intra- and extrahepatic CCA, with extrahepatic being the more common [1]. Global incidence of intrahepatic CCA specifically has increased dramatically in the last four decades, with poor overall survival (OS) and high rates of recurrence following surgical resection [2-4]. Although recurrence is high, there is evidence to suggest early repeat resection in select patients may lead to prolonged disease-free and OS, highlighting the need for appropriate follow-up [5-7]. However, there is scant data to support a specific schedule or modality for surveillance following surgery, and current guidelines are based on the schedule used in the phase III BILCAP trial [1,8].

Circulating tumor DNA (ctDNA) is a form of liquid biopsy that has been established as an effective tool in the prognosis and surveillance of various malignancies. Shed from dying tumor cells into the blood, ctDNA has the ability not only to detect minimal residual disease but also to inform further treatment decisions based on molecular profiling. Assays may be tumor-informed or tumor-agnostic, with the former requiring tissue sampling to evaluate detectable mutations present in an individual patient’s tumor [9]. CtDNA provides prognostic information after surgery, information on tumor heterogeneity, and a quantifiable risk of recurrence in a wide range of malignancies [10,11]. For example, in one study, the presence of ctDNA following curative intent resection of non-metastatic colorectal cancer (CRC) conferred a 25% decrease in five-year OS and a 47% decrease in five-year progression-free survival [11]. Indeed, much of the mature clinical data on ctDNA comes from the CRC. However, little data exists regarding the role of ctDNA in CCA, especially its role in surveillance. Here, we present a case of localized intrahepatic CCA that had recurred following standard-of-care therapy, with management guided by the use of ctDNA in conjunction with history, physical exam, laboratory work-up, and routine imaging.

## Case presentation

**Figure 1:**
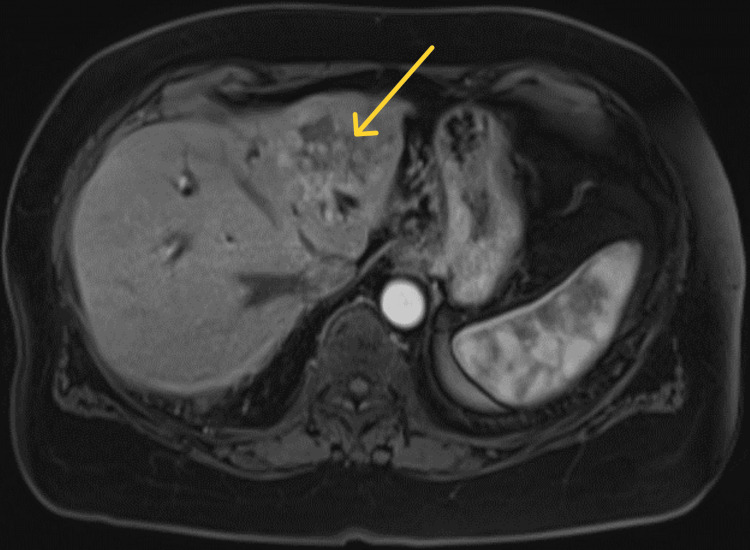
MRCP at diagnosis reveals a 5.7 x 5.2 cm left hepatic lobe mass MRCP: magnetic resonance cholangiopancreatography

**Figure 2:**
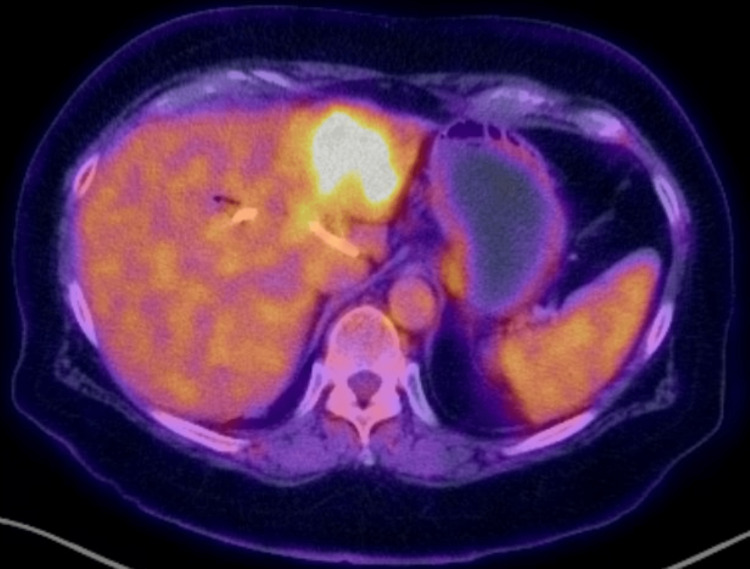
PET-CT with hypermetabolic activity in the left hepatic lobe extending into the hilum, consistent with biopsy-proven CCA PET-CT: positron emission tomography-computed tomography, CCA: cholangiocarcinoma

A 79-year-old female presented in December 2019 with a three-month history of postprandial epigastric abdominal pain, which worsened over the prior three weeks. She had developed jaundice 10 days prior to presentation with a total bilirubin of 7.1 mg/dL. Magnetic resonance cholangiopancreatography (MRCP) revealed a 5.7 x 5.2 cm left hepatic lobe mass with extension into the hepatic hilum obstructing the common hepatic duct; these findings were corroborated on subsequent positron emission tomography-computed tomography (PET-CT) (Figures 1-2). She underwent endoscopic retrograde cholangiopancreatography with plastic stent placement during that hospital stay and ultimately underwent left hepatectomy, extrahepatic bile duct resection, and Roux-en-Y right hepaticojejunostomy in January 2020. Brush cytology and surgical pathology were positive for invasive, poorly differentiated CCA with a final AJCC staging of pT3, NX, and M0. FGFR2 rearrangement was absent from fluorescence in-situ hybridization testing. Her tumor was microsatellite stable, and somatic mutations in BAP1, CCND1, FGF3, and FGF4 were detected. She completed eight cycles of adjuvant capecitabine per the BILCAP trial and was followed at our cancer center for active surveillance. Tumor-informed ctDNA was drawn in conjunction with CA 19-9 clinic visits every three months and computed tomography (CT) of the abdomen and pelvis every six months.

**Figure 3:**
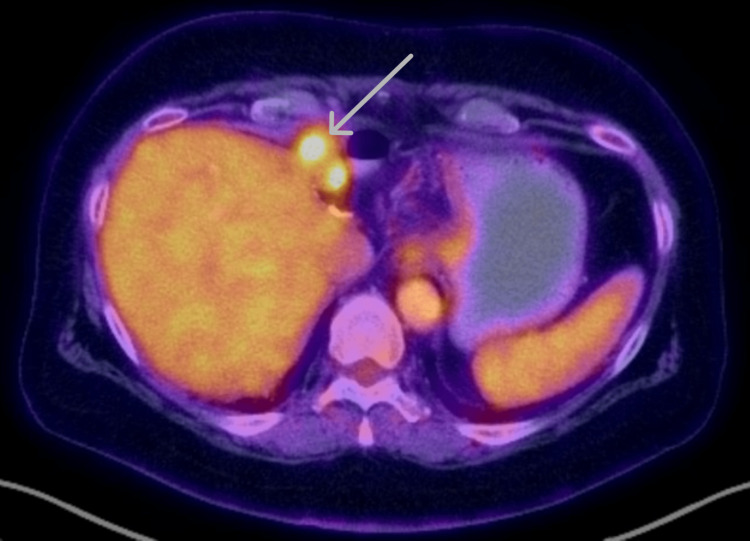
PET-CT with hypermetabolic foci at the hepatectomy margin over two years after original resection PET-CT: positron emission tomography-computed tomography

**Figure 4:**
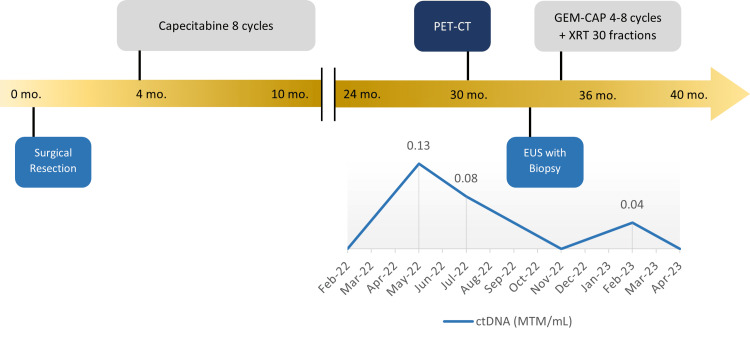
Clinical timeline from resection to recurrence and beyond CtDNA timeline follows in conjunction with interventions following the patient's recurrence in 2022 EUS: endoscopic ultrasound, GEM-CAP: gemcitabine-capecitabine, XRT: radiotherapy

Surveillance results remained negative until ctDNA returned positive at 0.13 MTM/mL (mean tumor molecules/milliliter) in May 2022, over two years after her original date of resection; interestingly, CT of the abdomen and pelvis did not show any evidence of recurrent disease at the time of ctDNA positivity. PET-CT performed two months later for evaluation of occult disease revealed a hypermetabolic focus near the hepatectomy margin, concerning local recurrence (Figure 3). She underwent endoscopic ultrasound (EUS) with a biopsy of the perihepatic anastomosis two months after her PET-CT, which revealed recurrent poorly differentiated CCA. We discussed options for therapy, and she began gemcitabine with capecitabine for four cycles, five months after her initial elevation in ctDNA, followed by concurrent capecitabine and radiation therapy (Figure 4). She is currently at her baseline functional status and is disease-free on radiologic and molecular studies, 40 months after her initial surgical resection.

## Discussion

CCA remains a disease in which surgical resection is the only potentially curative therapy to date. For the minority of those who are surgical candidates, residual disease remains a critical issue, with post-surgical five-year OS rates between 20% and 35% [4]. Beyond OS, recurrence is expected in over 50% of patients, with a median of 26 months. Early trials demonstrated a recurrence-free survival (RFS) for intrahepatic CCA of less than one year after curative-intent surgery, regardless of margin status [12]. By 2019, this had improved to 24 months with adjuvant capecitabine. Most recently, in the ASCOT trial, comparing adjuvant S-1 to observation following surgery for all biliary tract cancers, three-year RFS reached 62% [13-15].

As our understanding of CCA improves and patients begin to live longer, we must direct our attention to the surveillance of such a highly recurrent malignancy. Current National Comprehensive Cancer Network guidelines recognize that there is no data to support a specific schedule or tests for patients undergoing surgery. Thus, the algorithm for post-surgical follow-up is based upon the schedule used in the BILCAP trial, with serial CT or magnetic resonance imaging every three to six months for the first two years and every 6-12 months thereafter [1]. A 20-year single-center study published in 2022 followed the aforementioned guidelines and analyzed the role of early detection and intervention in recurrent CCA. Repeat surgical resection showed significant OS and RFS benefits compared to chemotherapy and best supportive care, and R0 resection outcomes were significantly better than R1 resections. In fact, none of the 14 patients who received R0 repeat resection went on to experience disease recurrence. However, only 17% of patients were able to undergo surgery [5]. Other studies have also shown the benefit of repeat resection [6-7], and in conjunction with the evidence that adjuvant chemotherapy also improves outcomes, it becomes clear that appropriate surveillance guidelines are needed.

The utility of ctDNA as a biomarker has been demonstrated repeatedly for use in pre-surgical recurrence risk, prognosis, and RFS estimates, most notably in CRC [10]. In comparison to current guidelines, serial ctDNA monitoring has been shown to predict CRC recurrence up to 16.5 months before CT imaging, with a median lead time of 8.7 and 9.8 months [16,17]. This length of time may be the difference in whether or not patients are able to undergo repeat resection and if R0 resection may be achieved, providing a chance at increased RFS [5-7]. For those patients who are not selected for repeat surgery, ctDNA has also been used to guide adjuvant chemotherapy use. For example, guidelines for the management of stage II CRC are not definitive and recommend analyzing the risks and benefits of adjuvant chemotherapy. The DYNAMIC trial utilized ctDNA to guide management, cutting adjuvant chemotherapy use nearly in half vs. standard management (15% vs. 28%) without sacrificing RFS (93.5% vs. 92.4% at two years) [18].

Similar prospective data are needed for CCA patients to inform clinical practice. Work in this space had been limited until as recently as 2021, when multicenter data demonstrated the practicality and utility of ctDNA use in hepatobiliary cancer. Similar to our patient, the presence of tumor-informed ctDNA correlated with both the stage of disease and the clinical response to therapy after surgery [19]. This study provided novel evidence of the feasibility of tumor-informed ctDNA, setting a benchmark for its continued study in patients with hepatobiliary cancer. These types of prospective data are needed to promulgate the utility of ctDNA in detecting minimal residual disease, monitoring recurrence, and guiding post-operative management to further inform clinical practice.

## Conclusions

CCAs are a subset of biliary tract cancers with a poor OS rate, a minority of patients who are candidates for curative-intent surgery, and high rates of recurrence thereafter. As surgical techniques improve and more adjuvant chemotherapeutic regimens are explored, appropriate surveillance guidelines are needed to keep up with prolonged survival. Our case demonstrates the utility of ctDNA as a biomarker in both detecting recurrence and guiding the management of intrahepatic CCA following curative-intent surgical resection. Positive ctDNA in our patient preceded both radiologic and clinical evidence of recurrence, consistent with previously published data, and has led to a favorable outcome without evidence of disease over three years from her initial surgical resection. Further studies using prospective data and clinical trials will allow us to identify patients who are at high risk of post-surgical recurrence, detect this recurrence earlier, and aim to improve relapse-free and OS in patients with CCA.
